# *Streptomyces iakyrus* TA 36 as First-Reported Source of Quinone Antibiotic γ–Rubromycin

**DOI:** 10.3390/molecules28165977

**Published:** 2023-08-09

**Authors:** Ivana Charousová, Miroslava Hlebová, Lukas Hleba, Juraj Medo, Joachim Wink

**Affiliations:** 1 Clinical Microbiology Laboratory, Unilabs Slovensko, s.r.o., J. Bellu 66, SK-03495 Likavka, Slovakia; ivana.charousova@unilabs.com; 2 Institute of Biotechnology, Faculty of Biotechnology and Food Sciences, Slovak University of Agriculture, Nitra, Tr. A. Hlinku 2, SK-94976 Nitra, Slovakia; 3 Department of Biology, Faculty of Natural Sciences, University of SS. Cyril and Methodius, Nám. J. Herdu 2, SK-91701 Trnava, Slovakia; 4 Microbial Strain Collection Group, Helmholtz Centre for Infection Research, Inhoffenstrasse 7, 38124 Braunschweig, Germany

**Keywords:** Streptomyces, MALDI–TOF MS, 16S rRNA, soil, γ-rubromycin

## Abstract

A wide range of bioactive compounds with potential medical applications are produced by members of the genus *Streptomyces*. A new actinomycete producer of the antibiotic γ-rubromycin, designated TA 36, was isolated from an alpine soil sample collected in Peru (Machu Picchu). Morphological, physiological and biochemical characteristics of the strain, together with data obtained via phylogenetic analysis and MALDI-TOF MS, were used for the correct identification of the isolate. The isolate TA 36 showed morphological characteristics that were consistent with its classification within the genus *Streptomyces*. Phylogenetic analysis based on 16S rRNA gene sequences showed that the TA 36 strain was most similar to *S. iakyrus* and *S. violaceochromogenes* with 99% similarity. Phylogenetic analysis together with the profile of whole cell proteins indicated that the strain tested could be identified as *S. iakyrus* TA 36. The crude extract Ext._5333_.TA 36 showed various effects against the tested organisms with strong antimicrobial activity in the growth of *Staphylococcus aureus* (Newman) (MIC value of 0.00195 µg/µL). HPLC fractionation and LC/MS analysis of the crude extract led to the identification of the quinone antibiotic γ-rubromycin, a promising antitumour and antibacterial antibiotic. To the best of our knowledge, there is currently no report on the production of γ-rubromycin by *S. iakyrus*. Therefore, this study suggests *S. iakyrus* TA 36 as the first-reported source of this unique bioactive secondary metabolite.

## 1. Introduction

Antibiotic resistance, as well as the need to find new antibiotics or new sources of existing antibiotics, is a very serious problem of this century [1,2]. In addition to antibiotic resistance, one of the leading causes of death in the world is cancer [3], and although progress has been made in the last two decades in the prevention of this disease and in the care of cancer patients, the number of cancer patients is still high [4,5]. The search for new anticancer drugs and antibiotics is therefore necessary, but the drug development process faces many obstacles (regulatory approval or high costs) [6]. For pharmaceutical companies, developing new products is therefore not a priority. Finding new sources of existing antibiotics or cancer drugs is therefore important [7]. 

Currently, there is a considerable amount of microbiological and pharmaceutical research being conducted for the control of human pathogens and the discovery of new species of microorganisms for the isolation of their antimicrobial agents [8,9]. Microorganisms, in particular those isolated from soil, are one of the most important natural sources of antimicrobial agents that are capable of inhibiting human pathogens [10,11]. Some of these microorganisms are also being explored for their production of antimicrobials with anticancer activities [12]. Among these, actinomycetes are a valuable group of prokaryotes that produce a number of secondary metabolites with a wide range of biological activities. These secondary metabolites include antibiotics, antitumour agents or immunosuppressants. For this reason, they are of economic and biotechnological importance [13,14]. Several thousand antibiotics that occur naturally in the environment have been isolated from actinomycetes [15], mainly from the genus *Streptomyces* [16]. Recently, fewer actinomycetes have been isolated from the general environment [17]. This is mainly due to the fact that unusual environments, such as forest and alpine soils, are still poorly understood and hold great promise for the discovery of bioactive compounds [18,19]

A remarkable amount of research has been devoted to the study of the bioactive quinone compounds produced by streptomycetes. Among related strains, *S. corcho-rusii* has been reported to produce resistomycin, an HIV-1 protease inhibitor [19,20]. Himalomycins A and B, two new quinone antibiotics with potent antibacterial activity, were isolated from the *Streptomyces* isolate B6921 [21,22], and whole komodoquinone A, a neuritogenic anthracycline, was isolated from the fermentation broth of *Streptomyces* sp. KS3 [23,24]. Because of their interesting biological activities and complex molecular architectures, the naturally occurring quinone rubromycin family has attracted the attention of a number of research groups over the last sixty years [25]. More recently, the authors Boumehira et al. [7] discovered that *Streptomyces* sp. ADR1, isolated from soil collected in the Algerian Sahara, produces two antibiotics: β- and γ-rubromycin. Rubromycins are characterised by a challenging molecular structure consisting of a naphthazarin moiety linked to an isocoumarin ring by a bis-benzannulated 5, 6-spiroketal system [26,27]. It has broad biological activities including antibacterial and anticarcinogenic activity [26,28,29,30] and activity against human telomerase-16 [31] and HIV-1 reverse transcriptase [32]. Studies show that there is a direct link between cancer and human telomerase, and that the spiroketane moiety of rubromycin is important in inhibiting it [7,33]. The results of the above-mentioned studies clearly demonstrate the high potential of soil reservoirs as an important source for isolating actinomycetes that can produce high-value antibiotic compounds. 

Therefore, this study aims to isolate and identify a *Streptomyces* strain as a new γ-rubromycin producer from alpine soil samples between two prominent peaks, Machu Picchu and Huayna Picchu, Peru. This strain, designated TA 36, was selected for its potential ability to produce antimicrobial molecules. Its antimicrobial and antifungal activities were determined in this study.

## 2. Results and Discussion

### 2.1. Morphological, Biochemical and Genetic Strain Characterisation

**Figure 1:**
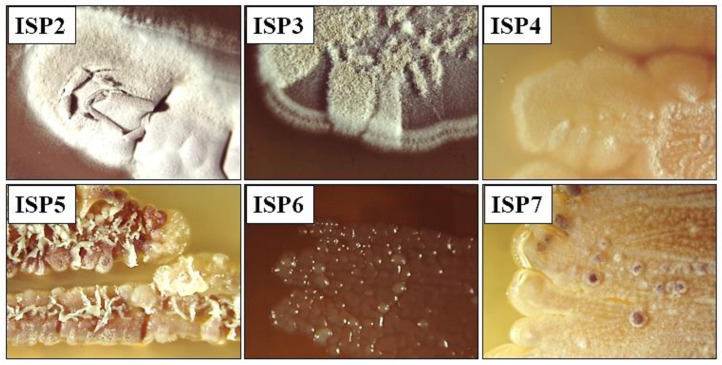
Culture characteristics of TA 36 strain on different ISP media.

Actinobacteria are of great biotechnological importance. Therefore, they are constantly being studied. These efforts to find new sources of biologically active substances have revealed that they are present in a wide variety of ecosystem sources, ranging from terrestrial to aquatic [34]. Furthermore, actinomycetes are capable of survival in extreme environments [35]. As a result, a wide variety of soil samples are constantly under investigation and new species or sources of microorganisms with potential antibiotic activity are being sought [36]. In this study, the TA 36 strain, an actinomycete with high inhibitory potential, was isolated from mountain soil (Machu Picchu, Peruvian Andes) as part of our routine screening programme of actinomycetes. This strain, designated TA 36, was found to be Gram-positive, aerobic and non-motile. It had a colony morphology typical of the genus *Streptomyces*. The strain TA 36 was subjected to polyphasic and molecular taxonomic studies in the present investigation. The strain was found to grow slowly. The strain formed a branched aerial mycelium. The spores were produced in spore chains of the Rectus-flexibilis type. After 3–4 days of incubation, sporulation was observed on the agar media. Strain TA 36 developed well on ISP2, ISP3, ISP4 and ISP7 media, but produced sparse aerial hyphae on ISP5 and ISP6 media (Figure 1). 

The TA 36 isolate grew over a temperature range of 25–37 °C, with the optimum temperature for growth recorded as 28–30 °C and no growth observed at 4 °C or above 37 °C. Growth occurred in the presence of 02.5% (*w*/*v*) NaCl (optimum range), but not at 5% and above NaCl. The pH range for growth was pH 5 to pH 7 (the optimal range was pH 6–7). Diffusible pigments were observed on the ISP6 medium. The substrate mycelium was olive to beige in colour. A fingerprint of enzymatic activities was obtained using API Coryne and API Zym test strips. 

Within the strain tested, significant enzymatic potential was detected. The TA 36 isolate showed high (>40 nmol) alkaline phosphatase, acid phosphatase, leucine arylamidase, beta-galactosidase and N-acetyl-glucoseamidase activity. It also showed moderate to low (3010 nmol) esterase C4, esterase lipase C8, valinarylamidase, trypsin, naphthol-AS-BI phosphohydrolase and alpha-mannosidase activity. Conversely, non-existent enzymes were detected as lipase C14, cystine arylamidase, chymotrypsin, α-galactosidase, beta-glucuronidase, α- and β-glucosidase and α-fucosidase. In the Api Coryne test, isolate TA 36 showed positive activity for α-glucosidase, gelatin hydrolysis, alkaline phosphatase and N-acetyl-β-glucosamidase, followed by moderate activity for nitrate reduction, esculin and urease production (Table 1). 

The differential characteristics between the TA 36 isolate and the most related type strains are shown in Table 2. 

**Figure 2:**
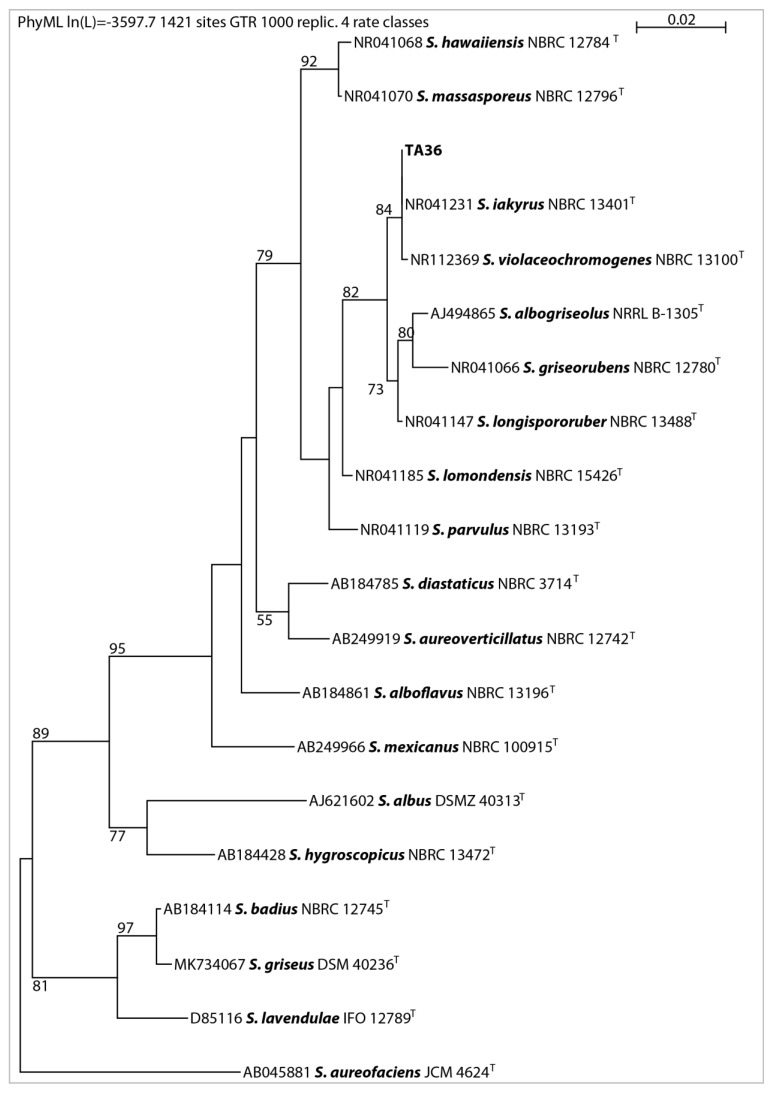
Phylogenetic position of isolate TA 36 among related Streptomycetes based on maximum likelihood analysis of 16S rRNA gene sequences, with numbers above branches indicating bootstrap support in % (only numbers higher than 50 are shown); ^T^-type culture.

**Figure 3:**
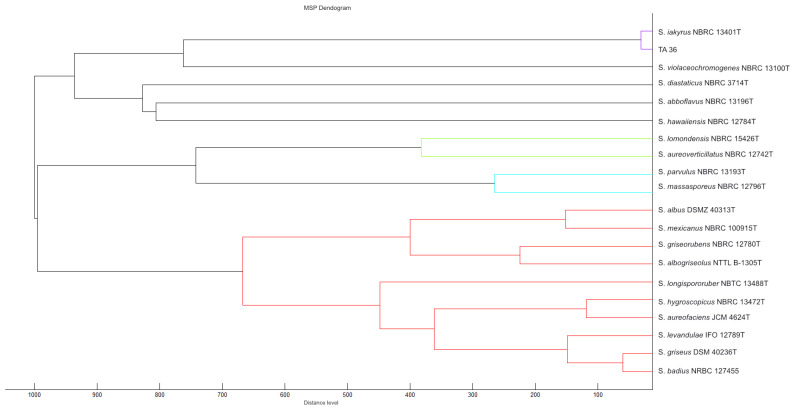
MSP dendrogram of tested *Streptomyces* strain and relatedness of TA 36 strain to *S. iakyrus* DSM 40482. The closest relationship of individual strains tested is indicated by colour differentiation of individual clusters.

In this work, 16S rRNA was sequenced. It was compared with the 16S rRNA sequences of previously described streptomycetes. The results showed that the almost-complete (1467 bp) 16S rRNA gene sequence of strain TA 36 was similar to the other members of the *Streptomyces* genus, in particular *S. violaceochromogenes* (NBRC13100T) (99% similarity), and it was identical to the sequence of *S. iakyrus* (NBRC 13401T). A comparison of the micromorphology of the strains showed that *S. violaceochromogenes* had Retinaculum apertum sporophores. *Streptomyces* TA 36 and *S. iakyrus* had a Rectus flexibilis shape. An extensive literature search revealed that *S. iakyrus* is capable of producing a wide range of antibiotic and antitumour agents including actinomycin [37] and iakirine [38]. *S. violaceochromogenes* produces arugomycin [39] and cinerubin [40] with antibacterial and antitumour activities. The phylogenetic tree topology, inferred via the maximum likelihood method based on the 16S rRNA gene sequences of the TA 36 strain and the closest related species of the genus *Streptomyces* (Figure 2), and the MSP dendrogram, constructed using previously described Streptomyces database spectra (Figure 3), were in agreement. The morphological, physiological and biochemical characteristics of the TA 36 strain, together with the data obtained from the phylogenetic and MSP analyses, indicated that the tested isolate could be identified as *S. iakyrus*. 

### 2.2. Evaluation of Antimicrobial Activity and Determination of Bioactive Compound

The crude extract of *S. iakyrus* (Ext._5333._TA 36) showed good antibiotic activity against the tested microorganisms. Gram-positive bacteria were more sensitive to the action of the extract compared with Gram-negative bacteria. The highest antimicrobial activity was shown by the extract against *S. aureus* (Newman), with an MIC value of 0.00195 µg/µL (Table 3).

Relatively high antimicrobial activity was registered against *B. subtilis* and *Micrococcus luteus* with minimum inhibitory concentrations of 0.0039 and 0.00781 μg/μL, respectively. The strongest antimicrobial activity of actinomycetes extracts against *B. subtilis* and *S. aureus* was also observed by Balachandar et al. [41]. The moderate antimicrobial activity was observed against *E. coli* (TolC) with an MIC value of 0.03125 μg/μL and *Chromobacterium violaceum* with an MIC value of 0.0625 μg/μL. The low antimicrobial activity was demonstrated against all tested fungal strains. The MIC ranged from 0.25 to 0.125 μg/μL. Our findings are in agreement with Gacem et al. [42]. The significant antibacterial activity of isolate TA 36 makes it a suitable candidate for further investigation of antagonistic activity against human pathogens. However, it was not clear what the nature of the antibacterial activity was. Therefore, the extract was subjected to HPLC fractionation and LC/MS analysis to determine which compound was active.

**Figure 4:**
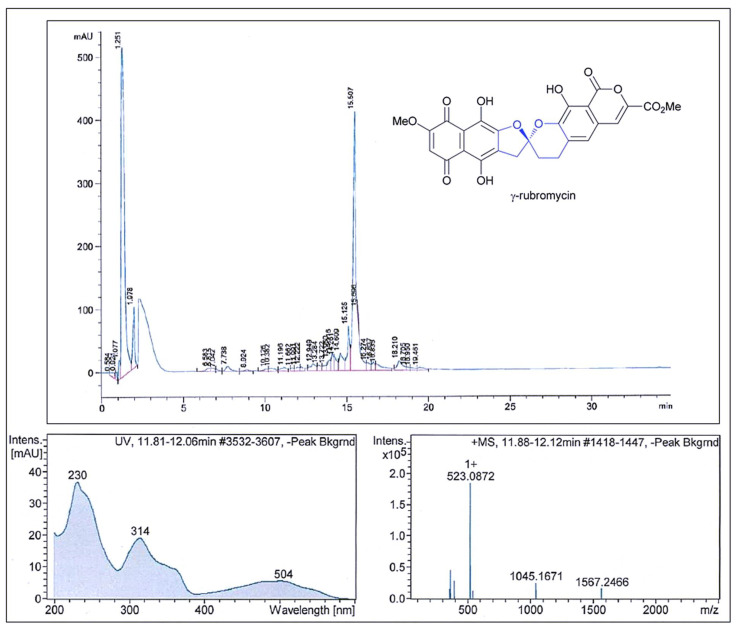
Comparison of the fractionation RP–HPLC chromatogram of γ–rubromycin, UV spectra with the max. at 314 nm and the ESI–HRMS spectrum showing the prominent ion clusters.

The results of the antimicrobial activity after the fractionation of Ext._5333_.TA 36 showed that the fractions had strong antimicrobial activity against *S. aureus*. The active fractions were found between retention times of 14.0 and 16.5 min based on the Peak Activity Correlation Test (Figure 4) of Ext._5333_.TA 36 extract.

The active peak, which appeared on the HPLC chromatogram during the retention time, showed UV-VIS maxima at 314 nm and an ESI-HRMS spectrum with significant ion clusters for [M − H_2_O + H]^+^ at *m/z* 523.0872. According to the available databases, this data set could be assigned to γ-rubromycin. Very similar data have been reported by Boumehira et al. [7], who studied the strain *Streptomyces* ADR1 as a potential source of β- and γ-rubromycin, which are anticancer antibiotic compounds. Two compounds were identified at the UV wavelength of 490 nm: Compound **1** was β-rubromycin (HRESIMS *m*/*z* 537.1024) and Compound **2** was γ-rubromycin (HRESIMS *m*/*z* 523.0869). For example, authors Harunari et al. [43] studied the culture extract of marine-derived *Streptomyces* sp. They found that this strain is capable of producing hyaluromicin, a new member of the rubromycin family of antibiotics. In their study, the extract was fractionated via reversed-phase column chromatography, followed by HPLC purification, and the data obtained showed the pseudomolecular ion [M + H]^+^ at *m*/*z* 604.1091. Their obtained value was higher than ours and they identified this compound as being hyaluromycin, which consists of a γ-rubromycin core structure possessing a 2-amino-3-hydroxycyclopent-2-enone (C_5_N) unit as an amide substituent of the carboxyl function. γ-Rubromycin belongs to a structurally related group of antibiotics—the rubromycins [38]. Rubromycins are potent antimicrobial agents. It has been reported that γ-rubromycin inhibits the growth of various bacteria and fungi at minimal concentrations (µM and nM) [26,44]. The genomes of the *Streptomyces* species contain a large number of clusters of genes encoding secondary metabolites. This has made them a rich source of compounds of industrial and clinical relevance [39,45]. The quinone antibiotic rubromycin, discovered by Brockmann and Renneberg [26], has the ability to selectively inhibit human immunodeficiency virus-1 (HIV-1) RNA-directed DNA polymerase (reverse transcriptase) (RT) activity [39]. To our knowledge, only the species *S. natalensis*, *Saccharopolyspora erythraea* [23] and *S. collinus* [26] have been reported as effective producers of rubromycins.

## 3. Material and Methods

### 3.1. Strain Identification and Characterisation

#### 3.1.1. Sample Collection and Isolation of the TA 36 Strain

Strain TA 36 was isolated from a soil sample collected in Peru (13°9′29.4″ S 72°32′47.1″ W) (2438 m above sea level) 70 km from Cusco, Peruvian Andes, using dilution agar plating. The soil sample was collected from a depth of 4–5 cm, placed in clean polyethylene bags and dried at room temperature for 3 days. The slightly acidic pH (5.9) of the soil was determined potentiometrically. One gram of the soil sample was mixed with 10 mL of sterile distilled water and stirred for 10 min to prepare a serial dilution. Streptomycetes were isolated using the spread plate technique on starch and casein medium [46]. Petri dishes were incubated at 30 °C for 7 days. The selected strain was cultured on International Streptomyces Project (ISP2) medium [28] at pH 7.0 at 28 °C for 10 days. It was then preserved in glycerol (30% *v*/*v*) and stored at −20 °C.

#### 3.1.2. Morphological Characterisation

The morphological characterisation consisted of a macroscopic characterisation and a microscopic characterisation. After 7–14 days of culture on ISP [28], the colours of the mature sporulating substrate and aerial mycelium of the TA 36 isolate were observed. Synthetic Suter’s medium [29] with or without tyrosine was used to detect melanin pigment. For the observation of spore chain morphology via light microscopy (OLYMPUS CX22LED, Japan), a well-grown agar plate containing glucose yeast medium (GYM medium) [47] was used. The observed morphological characteristics of the isolate were compared with the ‘Compendium of Actinobacteria’ provided by Dr Joachim Wink, Braunschweig, Germany, for presumptive isolate identification (https://www.dsmz.de/collection/catalogue/microorganisms/special-groups-of-organisms/compendium-of-actinobacteria, accessed on 11 June 2023).

#### 3.1.3. Physiological and Biochemical Tests

Using 12-well plates (BRAND, Washington, DC, USA), the ability of the strain to utilise 10 compounds as sole carbon sources for energy and growth was assessed on ISP9 medium after 7 days at 28 °C. Each source was added to the medium at a final concentration of 1% (*w*/*v*). The use of individual carbon sources was investigated according to Shirling and Gotlieb [28]. The effect of salt on growth was determined on the ISP9 medium supplemented with graded doses of sodium chloride (NaCl) (2.5, 5.0, 7.5 and 10% *w*/*v*) using six-well plates (BRAND, Washington, DC, USA). The maximum concentration of NaCl in the medium that allowed growth was determined. Growth at different temperatures (4, 10, 15, 25, 30, 37, 40, 45 °C) and pH (pH 2.0~10.0) was tested on the ISP2 plates. Commercial kits like ApiZym^®^ and ApiCoryne^®^ (bioMérieux, Craponne, France) were used to characterise the strain biochemically [48,49,50]. In order to carry out the Api^®^ tests, the culture was grown in a shaking flask with the GYM medium for a period of one week. The Api^®^ strips were incubated for 24 h at a temperature of 30 °C. The required reagents were added to each well after the incubation period. After 5 min, the strips were scored according to the manual criteria. 

#### 3.1.4. Genotypic Identification and Phylogenetic Analysis 

The 16S rRNA gene of strain TA 36 was sequenced for taxonomic classification in addition to morphological characteristics. The Spin Plant Mini Kit (Invisorb, Berlin, Germany) was used to extract genomic DNA. Nucleo-Spin^®^ Gel and PCR Clean-up-Kit (Macherey-Nagel, Düren, Germany) were used for purification of PCR products. The analysis procedure is described in Charousová et al. [51,52]. Sequences of type strains for the most similar species were downloaded from the NCBI nucleotide collection database (https://www.ncbi.nlm.nih.gov/nuccore, accessed on 11 June 2023). The phylogenetic tree was constructed via the maximum likelihood method using PhyML [53]. A general time reversible model was used for maximum likelihood analysis with optimised nucleotide equilibrium frequencies, optimised for site variation, and the best of NNI and SPR tree search. The initial tree was generated via BioNJ [54]. The sequence obtained was deposited under accession number OR197579.

#### 3.1.5. MALDI–TOF MS Analysis of Strain TA 36 

Intact proteins were isolated and extracted following Hleba et al. [55] with subsequent MALDI-TOF MS analysis. Extractions were performed on four randomly selected Petri dishes inoculated with purified TA 36 strain. After the extraction, 1 µL of the supernatant was transferred to a MALDI-TOF stainless steel plate (Bruker Daltonics, Bremen, Germany) in triplicate, by means of pipping. The samples were allowed to dry at room temperature. After drying, samples were coated with 1 µL matrix containing α-cyanohydroxycinnamic acid (Sigma Aldrich, St. Louis, MO, USA) diluted in 60% ethanol (60%, Sigma Aldrich, St. Louis, MO, USA), acetonitrile (50%, Sigma Aldrich, St. Louis, MO, USA) and trifluoroacetic acid (2.5%, Sigma Aldrich, St. Louis, MO, USA). The solvent was allowed to evaporate by drying at room temperature. All samples were analysed using a Microflex LT MALDI-TOF mass spectrometer (Bruker Daltonics, Bremen, Germany). The instrument was controlled via the FlexControl software (version 3.4) and operated in linear positive ion mode at 20 kV. Intact proteins were measured in the range of 2–20 kDa according to a standardised protocol established by Bruker Daltonics. *E. coli* (ref. 255343, Bruker Daltonics, Bremen, Germany) was used as a positive control standard. As a negative control, pure matrix solution was used. The spectra obtained via MALD-TOF MS were compared with the previously extensive local database of Streptococcal spectra described by Hleba et al. [55], where the authors prepared the local spectra database of DSMZ (German Collection of Microorganisms and Cell Cultures, Braunsweigg, Germany) Streptococcal strains. The MSP (Mean Spectra Projection) dendrogram for the clustering of *Streptomyces* and TA 36 strains was constructed using the appropriate software (MALDI Biotyper ver. 3.0, Bruker Daltonics, Bremen, Germany).

### 3.2. Extraction of Crude Extract and Antimicrobial Activity Screening

#### 3.2.1. Bacterial and Fungal Test Organisms Used in the Study 

Indicator microorganisms were grown overnight in the Mycosel broth [40] at 30 °C for the yeasts *Pichia anomala* (DSM 6766T) and *Candida albicans* (DSM 1665) and the filamentous fungus *Mucor hiemalis* (DSM 2656T), as well as in Mueller–Hinton (MH) broth (Merck, Darmstadt, Germany) at 30 °C in the case of Gram-positive bacteria: *Mycobacterium smegmatis* (ATCC 700084), *Micrococcus luteus* (DSM1790), *Staphylococcus aureus* (Newman) and *Bacillus subtilis* (DSM 10T), and Gram-negative bacteria: *Pseudomonas aeruginosa* (PA14), *Escherichia coli* (DSM 1116), *Chromobacterium violaceum* (DSM 30191T) and *Escherichia coli* (TolC). Then, the tested microorganisms were diluted with the culture medium to obtain an inoculum in a final concentration of 0.05 (bacteria) and 0.01 (yeasts) McFarland. The fungal inoculum of *M. hiemalis* (DSM 2656) was prepared in sterile distilled water to a final concentration of 1 × 10^6^ spores/mL. 

#### 3.2.2. Primary and Secondary Screening of Antimicrobial Activity

Prior to crude extract preparation, the TA 36 strain was cultured in 5333 medium (starch—15 g, yeast extract—4g, K_2_HPO_4_—1 g, MgSO_4_·_7_H_2_O—0.5 g, distilled water—1000 mL, pH—7). After five days of incubation, 20 mL of the culture was mixed with 20 mL of ethyl acetate (Sigma-Aldrich, USA). The mixture was shaken for 12 min. The sample was then centrifuged (9000 rpm, 10 min) and the supernatant containing the metabolite was transferred to a round bottom flask. A rotary evaporator (Heidolph instruments, Schwabach, Germany) was used to evaporate the ethyl acetate completely at 40 °C. After evaporation, the extract was prepared in accordance with Gacem et al. [42]. 

The primary screening was carried out according to the agar plug [56,57]. The broth microdilution method [44] in 96-well microplates (BRAND, Wertheim, Germany) was used for the secondary screening of the antimicrobial activity of the TA 36 strain. The dilution steps and the minimum inhibitory concentration (MIC) of the crude extract were observed by means of the inhibited wells (A-H). The more wells that were inhibited, the higher the activity of the tested extract was. For MIC evaluation, the crude extract was prepared to give final concentrations of 0.25 to 0.00195 µg/µL. The prepared 96-well plates were incubated at 37 °C for 24 h and the test fungi were incubated for 48 h. The values were obtained after 24 h via visual observation of the growth [52].

### 3.3. Fractionation of the TA 36 Crude Extract via HPLC and LC/MS Analysis

The selected extract showed high inhibitory potential against the Gram-positive bacterium *S. aureus* (Newman). Therefore, it was fractionated via HPLC (Agilent 1100 series with an X-Bridge C18 3.5 µm, 2.1 × 100 mm Column (Waters, Milford, CT, USA) and LC/MS analysis (Agilent 1200 series) with DAD detector (200–600 nm) in cooperation with a maXis UHR-TOF mass spectrometer (Bruker Daltonics, Billerica, MA, USA)). These methods have been described in detail previously in Charousová et al. [52]. Fractions (0.15 mL) from the HPLC column were collected into 96-well plates every 0.5 min. The fractions were then dried with nitrogen at 40 °C in a MiniVap (Porvair Sciences, Wales, UK) for 45 to 60 min. Then, 150 μL of *S. aureus* (Newman) culture in sufficient growth medium was added to each well. Due to the high inhibitory activity of the extract (visibly inhibited wells), the extract was applied to the LC-MS system. Peak/activity correlations were performed. The results were processed using the data analysis included in Compass software 4.1 (Bruker, Madison, WI, USA). Comparisons of molecular weights, bioactivity, UV spectra and retention times were used to identify the active compound.

## 4. Conclusions

The results of this study present more precise identification of strain TA 36 via the analysis of protein spectra using MALDI-TOF MS. The active isolate was identified as *S. iakyrus* by combining MALDI-TOF MS analysis with 16S rRNA sequencing and by studying the classical physiological, morphological and biochemical properties of the two most closely related strains. The present study demonstrated the strongest antimicrobial activity of Ext._5333_.TA 36 against *S. aureus* and good antibacterial activity against *B. subtilis* and *M. luteus*. Based on the peak data obtained via HPLC and LC-MS analysis, it was suggested that the active fraction against these pathogens was γ-rubromycin. This work provides evidence for a new producer of this antimicrobial compound, as there are no reports of γ-rubromycin production by *S. iakyrus* in the available literature. Further studies are now underway in our laboratories to purify the antibiotic γ-rubromycin itself, as well as to identify the other components present in the active Ext._5333_.TA 36.

## Figures and Tables

**Table 1:** Enzymatic characteristics of TA 36 strain.

Biochemical Test		TA 36
Api zym	Phosphatase alkaline	5
Api zym	Esterase (C4)	3
Api zym	Esterase lipase (C8)	3
Api zym	Lipase (C14)	1
Api zym	Leucin arylamides	4
Api zym	Valine arylamides	4
Api zym	Cystine arylamides	1
Api zym	Trypsin	2
Api zym	Chymotrypsin	1
Api zym	Phosphatase acid	5
Api zym	Naphthol-AS-BI-phosphohydrolase	3
Api zym	Alpha galactosidase	1
Api zym	Beta galactosidase	5
Api zym	Beta glucuronidase	1
Api zym	Alpha glucosidase	1
Api zym	Beta glucosidase	1
Api zym	N-acetyl-beta-glucoseamidase	5
Api zym	Alpha mannosidase	4
Api zym	Alpha fructosidase	1
Api coryne	Nitrate reduction	+
Api coryne	Pyrazinamide	−
Api coryne	Pyrrolidinyl arylamides	−
Api coryne	Alkaline phosphatase	+
Api coryne	Beta glucuronidase	−
Api coryne	Beta galactosidase	−
Api coryne	Alpha glucosidase	+
Api coryne	N-acetyl-beta glucoseamidase	+
Api coryne	Esculin	+
Api coryne	Urease	+
Api coryne	Gelatine (hydrolysis)	+
Api coryne	Glucose fermentation	+
Api coryne	L-arabinose fermentation	+
Api coryne	Xylose fermentation	−
Api coryne	Mannitol fermentation	+
Api coryne	Fructose fermentation	+
Api coryne	Sucrose fermentation	+
Api coryne	Raffinose fermentation	+
Api coryne	I-inositol fermentation	+
Api coryne	Rhamnose fermentation	+

Legend: determination of the enzymatic and fermentable capacities of TA 36 strain via Api Zym^®^ and Api Coryne^®^; 0 to 5: level of enzymatic expression by the strain; from absence to excellence expression. +: presence of enzyme activity, −: no enzyme activity.

**Table 2:** Different phenotypic characteristics of strain TA 36 and its phylogenetic neighbours of the genus Streptomyces.

Characteristics	Isolates		
	*Streptomyces* TA 36	*Streptomyces iakyrus* (NBRC 13401T)	*Streptomyces violaceochromogenes* (NBRC13100T)
Spore chain morphology	Rectus flexibilis	Rectus flexibilis	Retinaculum apertum
Aerial mass colour (ISP2–ISP7)	ISP2-4 grey olive, ISP5-7 sparse	ISP2-olive green, ISP3-4 granite grey, ISP5-7 none	ISP2-white, ISP3-5,7 grey, ISP6 none
Reverse mass colour (ISP2–ISP7)	ISP2-5-black olive, ISP6-7-black-sand yellow	ISP2-5-black olive, ISP6-7 beige	ISP2, 4, 7-brown, ISP3,5,6 yellow
Pigments	ISP6-signal brown	ISP6 grey brown	ISP5 red, ISP7 brown
Melanin	ISP6	+	+
Utilisation of carbon sources			
Glucose	+	+	+
Arabinose	+	+	−
Sucrose	(+)	+	−
Xylose	−	−	−
Inositol	+	+	−
Mannose	+	+	−
Fructose	(+)	+	−
Rhamnose	(+)	+	−
Optimal pH	6–7	7	7
Optimal temperature (°C)	28–30	28	28
NaCl tolerance (%)	2.5	2.5	7.5

Legend: + positive growth, − negative growth, (+) moderate growth, ISP2—yeast malt extract agar, ISP3—oatmeal agar, ISP4—inorganic salt starch agar, ISP5—glycerol–asparagine agar, ISP6—peptone yeast extract iron agar, ISP7—tyrosine agar.

**Table 3:** Antimicrobial activity of crude extract (Ext._5333._TA 36) during primary and secondary screening.

Indicator Test Microorganism	Primary Screening; Agar Plug Method (mm) ^a^	Secondary Screening; Broth Microdilution Method: MIC_5333_ (μg/μL)
*Bacillus subtilis* (DSM10)	35	0.0039
*Chromobacterium violaceum* (DSM30191)	17	0.0625
*Escherichia coli* (DSM116)	11	0.25
*Escherichia coli* (TolC)	19	0.03125
*Micrococcus luteus* (DSM1790)	24	0.00781
*Pseudomonas aeruginosa* (PA14)	12	-
*Mycobacterium smegmatis* (ATC700084)	22	0.03125
*Staphylococcus aureus* (Newman)	42	0.00195
*Mucor hiemalis* (DSM2656)	14	0.125
*Pichia anomalia* (DSM6766)	14	0.125
*Candida albicans* (DSM1665)	12	0.25

Legend: ^a^—diameter of the inhibition zone using the agar plug method, excluding the diameter of Streptomyces agar block (8 mm); MIC—minimum inhibitory concentration; MIC_5333_—minimum inhibitory concentration of crude extract Ext._5333_.TA 36.

## Data Availability

All data are available upon request on corresponding author.

## Sample Availability

Not available.
